# Supplementary figures and images for: MAF1 is a predictive biomarker in HER2 positive breast cancer
Source: PLoS One. 2023 Oct 6;18(10):e0291549. doi: 10.1371/journal.pone.0291549 (PMC10558074; doi:10.1371/journal.pone.0291549)

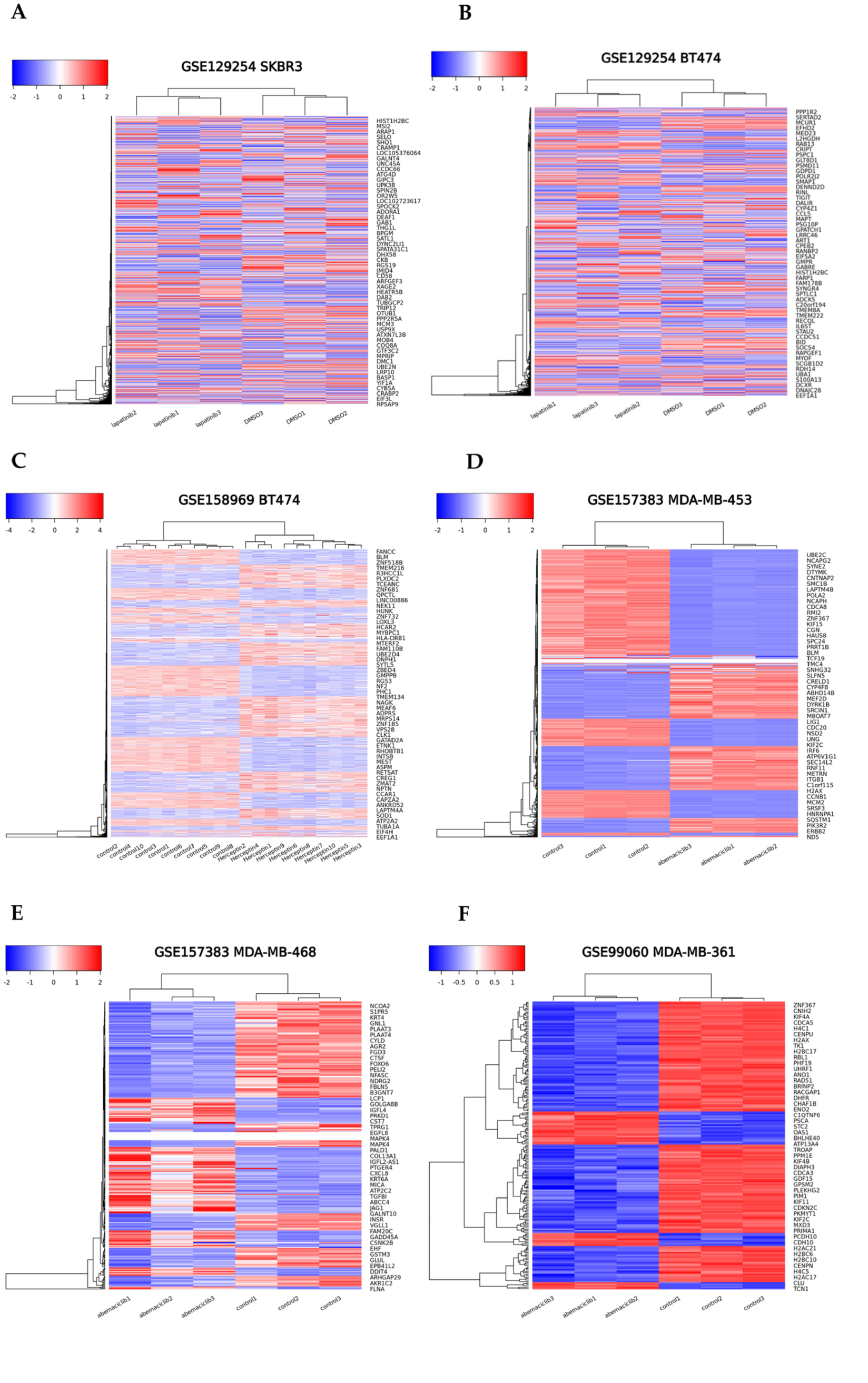

Supplement: S1 Fig — Heatmaps were generated in Galaxy using the heatmap2 tool [63]. Top differentially expressed genes (DEGs) are presented. Heatmaps for GSE129254 lapatinib-DMSO SKBR3 (A) and BT474 (B), and GSE158969 Herceptin-control in BT474 cells (C) top DEGs associated with Table 2 analyses. Table 3 associated heatmaps for GSE157383 MBA-MB-453 (D), GSE157383 MDA-MB-468 (E), and GSE99060 MDA-MB-361 (F) breast cancer cells treated with abemaciclib. (TIF) [file pone.0291549.s001.tif]

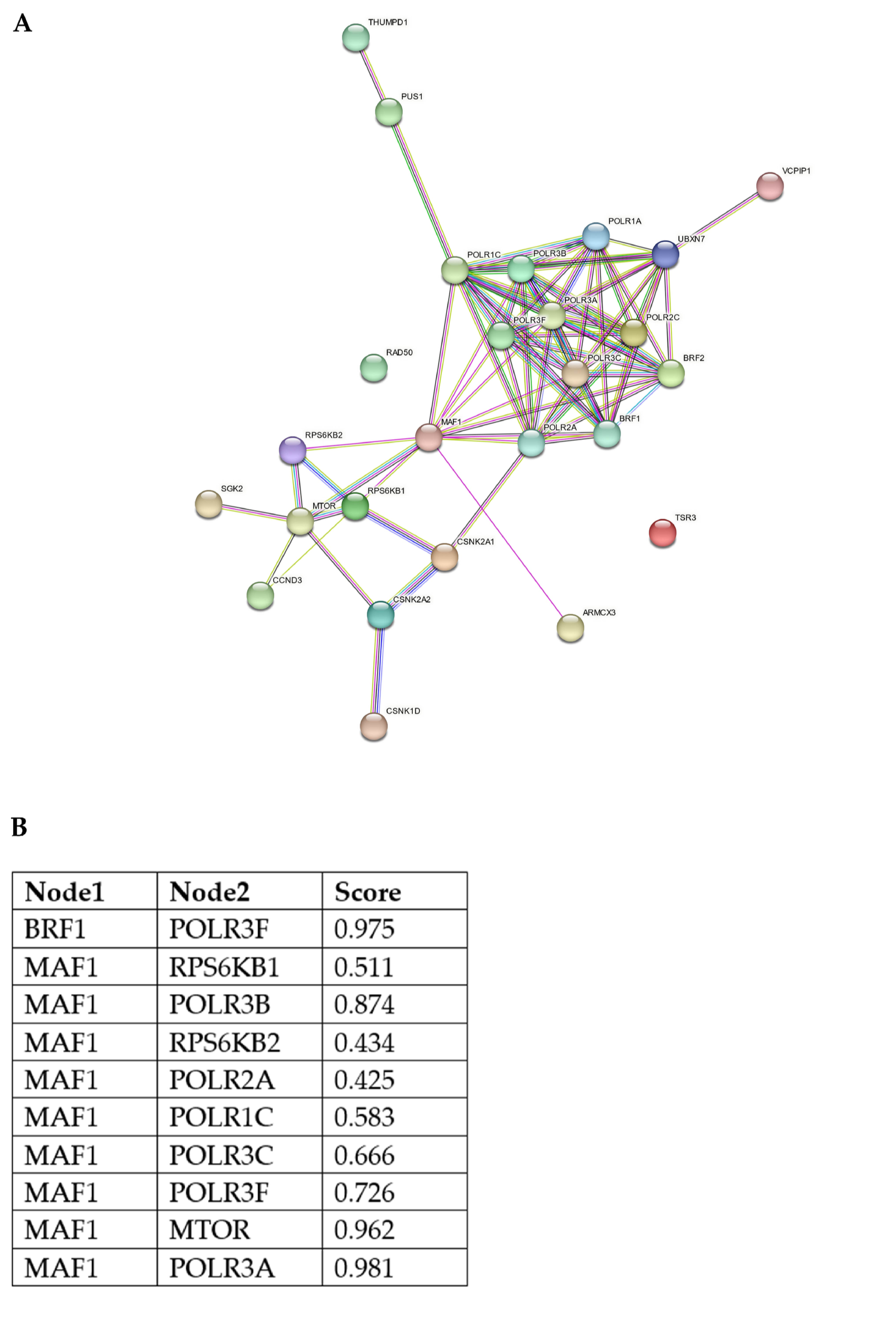

Supplement: S2 Fig — (A) Using String 11.5 [80] we identified protein-protein interactions (PPI) for MAF1. The PPI enrichment p-value < 1.0 x 10^–16^ suggests indicates that the proteins may be biologically connected. (B) Table of protein interaction scores, interaction score > 0.4 was applied. (TIF) [file pone.0291549.s002.tif]

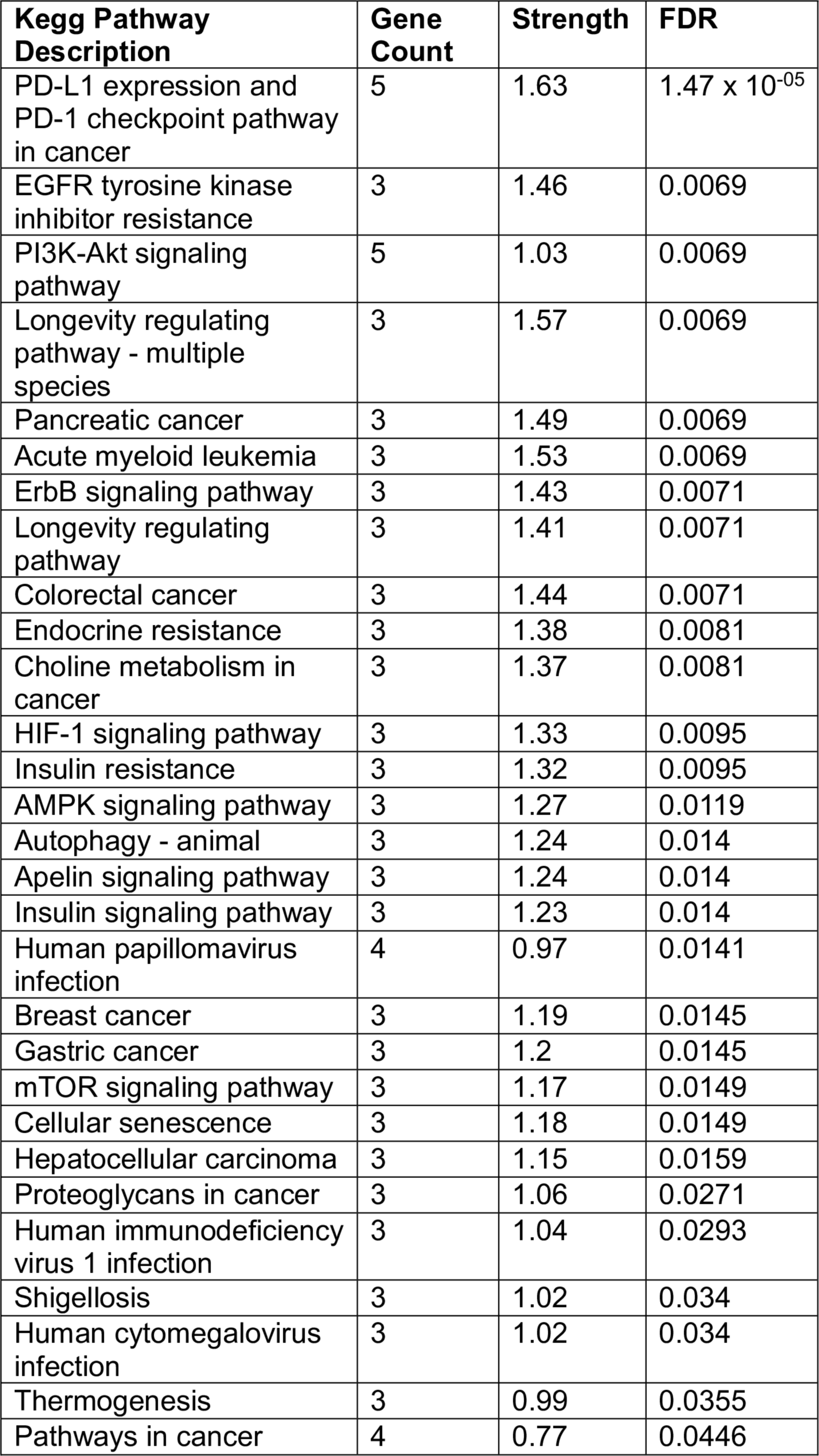

Supplement: S3 Fig — The most significant (FDR) Kegg pathways involved in the MAF1 network are presented. (TIF) [file pone.0291549.s003.tif]
